# Clinical risk factors associated with bone biopsy findings in CKD-associated osteoporosis: insights from the Brazilian Registry of Bone Biopsy (REBRABO)

**DOI:** 10.1007/s11657-026-01756-z

**Published:** 2026-08-14

**Authors:** Eduardo J. Duque, Lauren S. Lowe, Cinthia E. M. Carbonara, Rodrigo B. Oliveira, Thomas L. Nickolas, Rosilene M. Elias, Vanda Jorgetti, Luciene dos Reis, Aluizio B. Carvalho, Michael T. Yin, Rosa M. Moyses

**Affiliations:** 1 Nephrology Department, Laboratório de Fisiopatologia Renal (LIM16), Hospital das Clínicas, Faculdade de Medicina da Universidade de São Paulo, Av. Dr. Arnaldo, 455, 3º Andar, sala 3342, São Paulo, SP CEP 01246 903 Brazil; 2 Division of Nephrology and Hypertension, Center for Translational Metabolism and Health, Feinberg School of Medicine, Northwestern University, Chicago, IL USA; 3 Columbia University Irving Medical Center, New York, NY USA; 4 UNICAMP, Campinas, SP Brazil; 5 Division of Bone and Mineral Diseases, Department of Medicine, Washington University School of Medicine, St. Louis, MO USA; 6 Nephrology Department, Universidade Federal de São Paulo, São Paulo, SP Brazil

**Keywords:** Osteoporosis, Bone biopsy, Renal osteodystrophy, CKD, Mineral metabolism

## Abstract

***Summary*:**

Chronic kidney disease–associated osteoporosis (CKD-OP) is a highly prevalent—yet frequently underrecognized—condition in patients with advanced CKD. The assessment of bone turnover, mineralization, and volume remains challenging in clinical practice, often requiring bone histomorphometric analysis. Using data from the Brazilian Registry of Bone Biopsies (REBRABO), we identified clinical characteristics associated with these histomorphometric parameters. These findings may help clinicians better characterize the underlying bone phenotype and support a more individualized approach to the diagnosis and management of CKD-associated osteoporosis.

**Purpose:**

Chronic kidney disease–associated osteoporosis (CKD-OP) is characterized by impaired bone quality, and management of bone fragility in CKD partially relies on evaluating bone turnover (T), mineralization (M), and volume (V), through bone biopsy. However, bone biopsy data from large population-based cohorts are limited, making it challenging to identify clinical parameters that are associated with TMV patterns.

**Methods:**

This cross-sectional study is based on data from the Brazilian Registry of Bone Biopsy (REBRABO) and includes 374 iliac crest bone biopsies from adult patients with CKD5D (median age 51 y), collected between August/15 and December/21. Each biopsy was classified according to bone turnover (low/normal vs. high), mineralization (normal vs. abnormal), and trabecular volume (normal vs. low). Demographic and laboratory parameters were also analyzed.

**Results:**

No significant differences in TMV classification were observed based on ethnicity, sex, or diabetes status. Higher turnover was independently associated with younger age, hemodialysis modality, elevated parathyroid hormone (PTH), and alkaline phosphatase (ALP) levels, in a model adjusted for dialysis vintage, prior parathyroidectomy (PTX), and phosphate. Abnormal mineralization was independently associated with age, prior PTX, and lower serum calcium and phosphate, after adjustment for body mass index (BMI), PTH, and ALP. Low trabecular volume was independently associated with older age and lower BMI, in a model adjusted for PTH and history of fractures, and cannot be reliably inferred from biochemical markers. Notably, most patients with adynamic bone also presented with low trabecular volume.

**Conclusion:**

Our findings suggest that younger patients undergoing hemodialysis with elevated PTH and ALP levels may benefit from a more intensive approach to the management of hyperparathyroidism. In contrast, older and more frail patients with relatively low PTH levels may be more likely to exhibit low bone volume and turnover and could potentially benefit from therapies aimed at stimulating bone formation, such as anabolic agents.

## Introduction

Bone metabolism is continuously changing through the stages of chronic kidney disease (CKD) [1], and patients on dialysis have a fourfold higher hip fracture risk compared to the general population after adjustment for sex, age, and ethnicity [2]. CKD is associated with metabolic bone disease, characterized by biochemical alterations, vascular calcification, and skeletal abnormalities, known as renal osteodystrophy (ROD), which impairs bone quality and strength, increasing risk of fracture independent of bone density.

Osteoporosis is a systemic skeletal disorder characterized by weakened bone strength that results in increased risk of fracture. The concept of CKD-associated osteoporosis (CKD-OP) underscores the disruption of mineral and bone metabolism caused by CKD, characterized by impaired bone quality, not necessarily accompanied by low bone mineral density, together with alterations in bone turnover and disturbances in calcium, phosphate, and vitamin D homeostasis. In initial stages of CKD, the levels of fibroblast growth factor-23 (FGF-23) rise, functioning as a phosphaturic hormone to keep phosphate balance, inhibiting renal 1α-hydroxylase activity and fostering calcitriol [1,25(OH)_2_D_3_] deficiency [3]. Progressive hyperphosphatemia and calcitriol deficiency induce secondary hyperparathyroidism that increases fractional excretion of phosphate and disrupts serum calcium regulation by altering bone turnover and inducing bone fragility.

Patients with CKD are at higher risk of fractures, even when their bone mass is comparable to that of age- and sex-matched healthy individuals. To reduce the risk of fractures, therapeutic strategies must address CKD-specific mineral and bone disorder (CKD-MBD) abnormalities, such as secondary hyperparathyroidism, as well as low bone mass in order to mitigate defects in bone quality and the reduction in bone strength during CKD progression [4]. Current approach to managing bone fragility in CKD relies in part on assessment of bone quantity, estimated by bone mineral density, and bone quality by microarchitecture and tissue properties assessed by high-resolution quantitative computed tomography (HRpQCT), trabecular bone score, and bone biopsy. However, clinical and laboratory parameters that reliably predict metabolic bone disease in CKD patients are still lacking. As the epidemiological profile of ROD has changed with advances in treatment for CKD patients, we aimed to evaluate the risk factors that are associated with CKD-OP defined by bone biopsy, as well as the relationship between ROD type and trabecular bone volume.

## Methods

This retrospective cross-sectional subanalysis was conducted using data from the Brazilian Registry of Bone Biopsy (REBRABO). Between August 2015 and December 2021, 511 underwent bone biopsy. Of these, 137 were excluded due to reasons including normal kidney function, CKD in conservative care, previous kidney transplant, inadequate sample size or poor specimen quality. The final cohort comprised 374 patients who underwent iliac crest bone biopsy at the discretion of a nephrologist and had complete clinical, laboratory, and imaging data recorded in the REBRABO database.

Bone biopsy had at least one of the following indications: unexplained hypercalcemia or hyperphosphatemia, suspicion of aluminum accumulation, persistent bone pain, pathologic bone fractures, evaluation before starting bisphosphonate therapy or enrolling in parathyroidectomy (PTX) list, and research protocol. The exclusion criteria were as follows: refusal to sign the informed consent form, age < 18 years, estimated creatinine clearance ≥ 60 mL/min, current kidney transplantation, and bone biopsy indicated by a specialty other than nephrology.

Demographic and laboratorial data were collected from electronic charts and included age, sex, race, body mass index (BMI), history of diabetes mellitus and/or hypertension, dialysis method, previous fracture, major adverse cardiovascular events (MACE), dyslipidemia, smoking, previous PTX, levels of calcium, phosphate, albumin, 25-hydroxy(OH)vitamin D, alkaline phosphatase (ALP) and parathyroid hormone (PTH), use of calcium salts, sevelamer, vitamin D analogues, and calcimimetics.

Intact PTH (reference range, RR 15–65 pg/mL) and serum 25-OH vitamin D (RR = 30–60 ng/mL for risk groups, including CKD patients) were measured by chemiluminescent immunoassay. Serum total calcium (RR = 8.4–10.2 mg/dL), ALP (RR = 35–104 U/L), serum phosphate (RR = 2.3–4.5 mg/dL), and albumin (RR = 3.4 to 5.4 g/dL) were measured using colorimetric assay.

Bone samples were obtained by transiliac bone biopsy after double tetracycline labeling (20 mg/kg/day) administered in two courses of 3 days each, separated by a 10-day interval. The biopsy was performed 3–5 days after the last tetracycline dose and within 1 month of blood sampling. Undecalcified bone specimens were embedded in methyl methacrylate and processed according to standard histological procedures. For histological studies, undecalcified 5-µm-thick sections were stained with 0.1% toluidine blue; pH 6.4 and unstained 10-µm-thick sections were obtained for analysis of dynamic parameters under fluorescent microscopy.

Bone histomorphometric analysis was performed using a semiautomatic system (Osteomeasure software, Osteometrics Inc., Atlanta, GA, USA). Parameters were reported according to the nomenclature and abbreviations recommended by the American Society for Bone and Mineral Research (ASBMR) Histomorphometry Nomenclature Committee [5], and reference values are expressed as M (mean estimate as obtained by random effects model), and CI (95% confidence interval for mean estimate as obtained by random effects model).

The following parameters were evaluated:

- Static structural parameters: bone volume (BV/TV; %), defined as the volume occupied by mineralized and unmineralized trabecular bone, expressed as a percentage of the total volume occupied by bone marrow and trabeculae—reference value, 20.46% (95% CI 19.55–21.36); trabecular thickness (Tb.Th; μm), defined as the mean thickness of trabeculae, expressed in microns—reference value, 143.36 μm (95% CI 135.20–151.53); trabecular separation (Tb.Sp; μm), defined as average distance between the trabeculae, expressed in microns—reference value, 639.1 μm (95% CI 578.0–700.3); and trabecular number (Tb.N;/mm), expressed as the number of bone trabeculae per millimeter of tissue evaluated (it is given by the ratio between the number of trabeculae and their thickness—reference value, 1.5/mm (95% CI 1.3–1.6).
- Bone formation parameters: osteoid volume (OV/BV; %), defined as the volume occupied by non-mineralized bone (osteoid matrix) in relation to trabecular volume (mineralized and non-mineralized)—reference value, 2.22% (95% CI 1.83–2.62); osteoid thickness (O.Th; μm), defined as the thickness of the osteoid matrix, expressed in microns—reference value, 9.87 μm (95% CI 8.83–10.91); osteoid surface (OS/BS; %), expressed as the percentage of the trabecular surface covered by osteoid matrix—reference value, 14.64% (95% CI 13.55–15.73); and osteoblast surface (Ob.S/BS; %), expressed as the percentage of the trabecular surface covered by osteoblasts in relation to total trabecular surface—reference value, 3.62% (95% CI 2.04–5.20).
- Bone resorption parameters: eroded surface (ES/BS; %), expressed as the percentage of the trabecular surface that presents gaps in bone resorption, with or without osteoclasts—reference value, 4.3% (95% CI 3.67–4.93); and osteoclast surface (Oc.S/BS; %), expressed as the percentage of the trabecular surface that presents osteoclasts—reference value, 1.04% (95% CI 0.60–1.49).
- Dynamic parameters**:** mineralizing surface (MS/BS; %), expressed as percentage of trabecular surface covered with tetracycline double labels and one-half of tetracycline single labels in relation to total trabecular surface—reference value, 7.73% (95% CI 6.81–8.65); mineral apposition rate (MAR; µm/day), defined as the amount of mineral deposited in 24 h between two tetracycline labels—reference value, 0.6 µm/day (95% CI 0.57–0.64); bone formation rate (BFR/BS; μm^3^/μm^2^/year), defined as the product of the MAR and the MS/BS, expressed as the rate of mineralized bone per 24-h period—mean reference value, 0.059 μm^3^/μm^2^/day (95% CI 0.044–0.075); and mineralization lag time (Mlt; days), defined as the interval between deposition and mineralization of the osteoid matrix—reference value, 27.29 days (95% CI: 16.94–37.63).

Bone biopsies were classified according to the TMV system, which provides a clinically relevant description of the underlying bone pathology as assessed by histomorphometry. Turnover was defined low when the BFR/BS was less than one deviation from the mean and high when it was one deviation above the mean, using double tetracycline labeling [6]. Abnormal mineralization was diagnosed when osteoid thickness (O.Th) was greater than the upper limit of the reference value and the Mlt greater than 50 days. Low trabecular bone volume was defined as BV/TV below the lower limit of normal [7].

Renal osteodystrophy (ROD) was classified, as previously described [8], into high-turnover disease, including osteitis fibrosa (OF) and mixed bone disease (MD), the latter characterized by defective mineralization, and low-turnover disease, including adynamic bone disease (AB), when mineralization was preserved, and osteomalacia (OM), characterized by low bone turnover and defective mineralization [7, 9].

### Statistical analysis

Descriptive statistics for continuous variables were summarized using mean and standard deviations (SD) or as medians and interquartile ranges (IQR). Categorical variables are expressed as absolute values (*n*) and percentages (%). Data normality was tested by the Shapiro-Wilk test. Unpaired* t*-tests/Mann-Whitney tests (continuous variables) and chi-square/Fischer’s exact test (categorical variables) were used to analyze differences between groups according to TMV status, depending on Gaussian or non-Gaussian distribution. A multivariate stepwise linear regression and logistic regression were performed to identify the independent determinants of low trabecular bone volume, high turnover, and altered mineralization. All variables that reached statistical significance in the univariate analysis were included in the model of the multiple regression analysis. Statistical analyses were performed using SPSS 22.0 (SPSS Inc., Chicago, IL). *P* values < 0.05 were statistically significant.

## Results

The cohort comprised 181 women and 193 men, with a median age of 51 years (range, 18–79 years). Median PTH was 233 (63–780) pg/mL, phosphate 4.9 (3.9–6.2) mg/dL, calcium 9.3 (8.6–9.9) mg/dL, ALP 119 (79–221) U/L, and 25-OH vitamin D 29 (21–38) ng/mL. Calcium salts were being used in 95 (25%), sevelamer in 229 (61%), vitamin D analogues in 98 (26%), and calcimimetics in 95 (25%) patients.

According to histomorphometric criteria, patients were divided by bone turnover (low/normal vs. high), mineralization (normal vs. abnormal), and trabecular volume (normal vs. low) status. Table 1 shows bone histomorphometric characteristics. Abnormal mineralization presented with lower BV/TV, reduced trabecular thickness, and decreased osteoblast surface. As expected, formation and resorption parameters were higher in patients with high turnover, in contrast to decreased eroded and osteoclastic surface in those with abnormal mineralization. No other significant difference was observed between static parameters.

**Table 1:** Histomorphometric characteristics according to TMV status

Histomorphometry	Turnover			Mineralization			Volume		
	Low/normal	High	*p*	Normal	Abnormal	*p*	Normal	Abnormal	*p*
Static parameters									
BV/TV,%	19.36 (14.93–24.61)	21.72 (16.6–29.2)	0.07	22.52 (17.37–29.21)	18.82 (14.9–24.6)	**0.034**	28.9 (25.2–32)	16.54 (13.77–19.31)	** < 0.001**
Tb.Th, µm	121.3 (105.2–140.7)	129.1 (112.7–139.4)	0.44	132.1 (115.8–145.2)	119.9 (100.8–135)	**0.002**	136.1 (123.3–159.8)	115.7 (101.7–130.7)	** < 0.001**
Tb.Sp, µm	510.4 (387.7–590.3)	445.6 (329–605)	0.07	445.6 (345–607)	513.1 (372.5–597)	0.27	363.5 (302.5–391.1)	581.2 (510.2–685.9)	** < 0.001**
Tb.N,/mm	1.6 (1.33–1.94)	1.76 (1.37–2.1)	0.14	1.71 (1.36–2.03)	1.55 (1.34–1.97)	0.58	1.99 (1.8–2.3)	1.435 (1.225–1.613)	** < 0.001**
Formation									
OV/BV, %	0.77 (0.43–1.73)	3.46 (1.59–7.33)	** < 0.001**	1.72 (0.89–4.63)	1.38 (0.59–4.98)	0.24	1.75(0.67–4.84)	1.605 (0.62–4.05)	0.62
O.Th, µm	6.12(4.87–7.59)	8 (6.40–11.38)	** < 0.001**	7.45 (6.15–10.04)	7 (4.88–8.71)	0.06	7.62 (6.67–10.06)	6.275 (4.91–8.35)	**0.01**
OS/BS, %	7.95 (5.90–14.82)	25 (16.4–46.2)	** < 0.001**	17.75 (10.43–33.11)	12.9 (6.71–32.24)	0.18	18.58 (7.6–31.6)	14.4 (7.60–31.44)	0.69
Ob.S/BS, %	1.53 (1.06–2.43)	7.3 (4.3–14.2)	** < 0.001**	5.27 (1.99–10.45)	2.35 (1.23–5.77)	**0.002**	5.43 (1.52–9.08)	2.775 (1.29–5.83)	0.13
Reabsorption									
ES/BS, %	2.83 (1.95–3.9)	6.98 (4.34–9.52)	** < 0.001**	5.87 (3.72–8.93)	3.2 (2.13–4.99)	** < 0.001**	4.85 (2.86–8.03)	3.65 (2.28–6.99)	0.11
Oc.S/BS, %	0.12 (0.06–0.28)	0.56 (0.28–1.39)	** < 0.001**	0.475 (0.11–1.30)	0.2 (0.08–0.45)	**0.004**	0.29 (0.095–0.735)	0.285 (0.11–0.73)	0.82
Dynamic parameters									
MS/BS, %	3.88 (1.53–5.58)	9.48 (5.13–13.75)	** < 0.001**	8.56 (4.49–13.29)	3.08 (1.40–6.29)	** < 0.001**	7.53 (4.03–13.22)	4.49 (1.59–8.39)	**0.001**
MAR, µm/day	0.59 (0.47–0.71)	0.86 (0.62–1.11)	** < 0.001**	0.73 (0.59–1.05)	0.615 (0.45–0.75)	**0.006**	0.72 (0.5–1.04)	0.66 (0.48–0.86)	0.25
BFR/BS, µm^3^/µm^2^/day	0.02 (0.01–0.03)	0.07 (0.02–0.12)	** < 0.001**	0.06 (0.03–0.12)	0.01 (0–0.03)	** < 0.001**	0.04 (0.02–0.09)	0.03 (0.01–0.06)	**0.044**
Mlt, days	41.5 (19.8–81.1)	40 (13.45–90.3)	0.60	19.84 (10.67–43.75)	61.77 (42.8–220)	** < 0.001**	30.05 (14.35–57.94)	50.4 (19.85–97.38)	0.08
Medullary fibrosis									
Fb.V/TV, %	0.03 (0–0.04)	0.11 (0.01–0.47)	** < 0.001**	0.09 (0.02–0.38)	0.03 (0–0.07)	** < 0.001**	0.08 (0.01–0.475)	0.03 (0–0.08)	**0.009**

Data presented as median (IQR). Bold data indicate significance (*p *<0.05)

ALP alkaline phosphatase, PTH parathyroid hormone, BV bone volume, TV trabecular volume, Tb trabecular, Th thickness, Sp separation, N number, OV osteoid volume, OS osteoid surface, BS bone surface, ES eroded surface, Oc.S osteoclast surface, MS mineralizing surface, MAR mineral apposition rate, BFR bone formation rate, Mlt mineralization lag time, Fb fibrosis

**Fig. 1:**
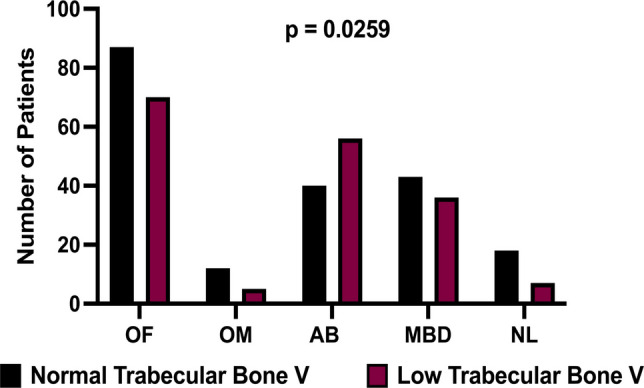
Prevalence of normal and low trabecular bone volume according to ROD type. Osteitis fibrosa (OF), osteomalacia (OM), adynamic bone (AB), mixed bone disease (MBD), normal (NL). Significant difference in distribution (chi-square, *p* <.05)

Based on histomorphometry, OF (*n* = 157, 42%) was the most frequent type of ROD, followed by AB (*n *= 96, 26%), MB (*n* = 79, 21%), and OM (*n* = 17, 4.5%). Normal histology or mild bone disorder were found in 6.5%. Low trabecular bone volume was present in 44% of patients with OF, 45.5% with MB, 58.3% with AB, and 29% with OM (Fig. 1).

**Fig. 2:**
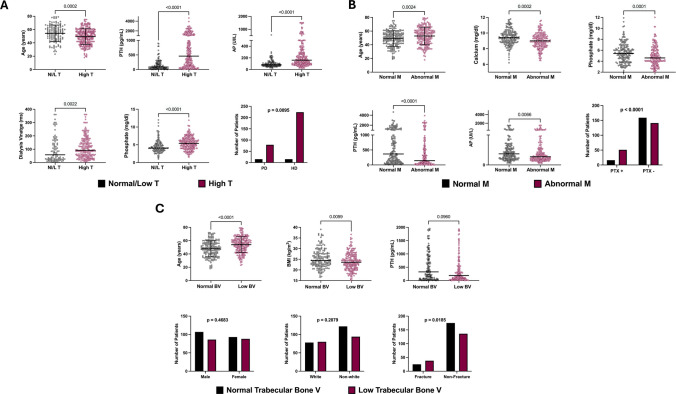
Distribution of clinical and biochemical characteristics in patients with normal/low vs high turnover (T) (**A**), normal vs abnormal mineralization (M) (**B**), and normal vs low trabecular volume (V) (**C**). Significant difference in distribution (chi-square, *p* <.05)

Clinical characteristics are summarized in Table 2 and Fig. 2. No differences were observed by ethnicity, sex, or history of diabetes, according to TMV status. While abnormal mineralization and low trabecular volume were observed in older patients, high turnover was more commonly found in younger patients. Patients with higher turnover were more likely to be receiving hemodialysis rather than peritoneal dialysis. Longer dialysis vintage and higher phosphate, PTH, and ALP levels were also observed in this group. Patients with abnormal mineralization had lower levels of calcium, phosphate, ALP, and PTH and were more likely to have undergone prior parathyroidectomy and to be taking calcium salts.

**Table 2:** Demographic and biochemical characteristics according to TMV status

	Turnover			Mineralization			Volume		
	Low/Normal 123	High 248	*p*	Normal 175	Abnormal 192	*p*	Normal 200	Low 174	*p*
Age, ys	54.3 ± 12.5	49.3 ± 12	** < 0.001**	48.9 ± 11.4	52.8 ± 12.8	**0.002**	47.7 ± 12	53.9 ± 12	** < 0.001**
BMI, mg/kg^2^	24.2 (22–27)	24.1 (22–27)	0.69	24.4 (22–27)	23.6 (21–26)	0.09	24.4 (22–28)	23.6 (21–27)	**0.005**
Dialysis Vintage, months	58 (23–144)	92 (48–157)	**0.002**	84 (38.5–144)	96 (36–168)	0.34	84 (42–144)	84 (36–156)	0.70
Female, *n* (%)	60 (48.8%)	118 (47.6%)	0.83	82 (46.9%	95 (49.5%)	0.61	93 (46.5%)	88 (50.6%	0.43
White, *n* (%)	57 (46.3%)	98 (39.5%)	0.21	73 (41.7%)	79 (41.1%)	0.91	78 (39%)	80 (46%)	0.17
HD/PD, *n* (%)	79/15(84/16%)	224/15 (93.7/6.3%)	**0.005**	143/14 (91.1/8.9%)	159/16 (90.9/9.1%)	0.94	159/17 (90.3/9.7%)	144/14 (91.1/8.9%)	0.80
Previous fracture, *n* (%)	17 (13.8%	45 (18.1%)	0.29	25 (14.3%)	37 (19.3%)	0.20	25 (12.5%)	38 (21.8%)	**0.016**
Diabetes, *n* (%)	21 (17.1%)	35 (14.1%)	0.45	27 (15.4%)	29 (15.1%)	0.93	31 (15.5%)	26 (14.9%)	0.88
Arterial hypertension, *n* (%)	97 (78.9%)	199 (80.2%)	0.75	140 (80%)	154 (80.2%)	0.96	172 (86%)	126 (72.4%)	**0.001**
Dyslipidemia, *n* (%)	35 (28.5%)	28 (11.3%)	** < 0.001**	27 (15.4%)	34 (17.7%)	0.56	34 (17%)	30 (17.2%)	0.95
Smoking, *n* (%)	14 (11.4%)	15 (6%)	0.07	11 (6.3%)	17 (8.9%)	0.35	20 (10%)	9 (5.2%)	0.08
Previous MACE, *n* (%)	9 (7.3%)	25 (10.1%)	0.38	18 (10.3%)	16 (8.3%)	0.52	17 (8.5%)	6 (3.4%)	1.0
Previous PTX, *n* (%)	30 (24.4%)	37 (14.9%)	**0.02**	16 (9.1%)	51 (26.6%)	** < 0.001**	36 (18%)	31 (17.8%)	0.96
Calcium salts, *n* (%)	33 (26.8%)	61 (24.6%)	0.64	30 (17.1%)	64 (33.3%)	** < 0.001**	54 (27%)	41 (23.6%)	0.44
Sevelamer, *n* (%)	47 (38.2%)	182 (73.4%)	** < 0.001**	128 (73.1%)	100 (52.1%)	** < 0.001**	124 (62%)	105 (60.3%)	0.74
Active vitamin D analogues, *n* (%)	24 (19.5%)	74 (29.8%)	**0.03**	44 (25.1%)	53 (27.6%)	0.59	55 (27.5%)	43 (24.7%)	0.54
Cinacalcet, *n* (%)	11 (8.9%)	84 (33.9%)	** < 0.001**	60 (34.3%)	35 (18.2%)	** < 0.001**	55 (27.5%)	40 (23%)	0.32
Vitamin D supplement, *n* (%)	29 (23.6%)	36 (14.5%)	**0.03**	27 (15.4%)	38 (19.8%)	0.27	29 (14.5%)	36 (20.7%)	0.11
Biochemical parameters									
Calcium (mg/dL)	9.2 (8.5–9.8)	9.3 (8.6–9.9)	0.36	9.4 (8.8–10.1)	9 (8.4–9.7)	** < 0.001**	9.2 (8.6–9.8)	9.3 (8.6–10)	0.50
Phosphate (mg/dL)	4.1 (3.4–5.3)	5.4(4.1–6.5)	** < 0.001**	5.4 (4–6.5)	4.6 (3.7–6)	** < 0.001**	5.1 (3.9–6.3)	4.8 (3.8–6.1)	0.16
PTH (pg/mL)	86.5(31.3–222)	460 (124–1110)	** < 0.001**	365.5 (112–1086)	142 (41.1–531)	** < 0.001**	325.5 (67.5–928)	189 (58.3–567)	0.09
ALP (U/L)	82 (62–116)	160.5 (92.2–287)	** < 0.001**	148 (88–246)	109 (73–203)	**0.006**	118 (73–217)	126 (81–234)	0.27
Albumin (g/dL)	3.8 (3.4–4.2)	4 (3.8–4.3)	**0.02**	4 (3.7–4.2)	4 (3.5–4.2)	0.66	4 (3.7–4.2)	4 (3.5–4.2)	0.49
25OH vitamin D (ng/mL)	28.1 (21.8–37.1)	30.3 (20.3–38.4)	0.67	29.1 (20–37.8)	28.4 (22–38)	0.96	29.6 (20.8–37.9)	28.4 (21–38.6)	0.77
Hemoglobin (g/dL)	11.6 (10.6–13.6)	11.4 (10.1–12.8)	**0.04**	11.4 (10.1–13.2)	11.5 (10.5–12.8)	0.56	11.5 (10.1–13)	11.6 (10.3–13)	0.60
Ferritin (ng/mL)	388 (209–674)	352 (185–742)	0.40	423.5 (201–805.9)	346 (173–647)	0.06	350 (198–679)	390.5 (187–795)	0.37
Bicarbonate (mmol/L)	23 (21.2–25.6)	22.8 (20.6–25)	0.51	23 (21–25)	22.7 (20.1–25)	0.52	22.3 (20–25)	23.8 (21.3–25.7)	0.08

Data presented as mean ± SD, median (IQR), or *n* (%). Bold data indicate significance (*p *<0.05)

BMI body mass index, HD hemodialysis, PD peritoneal dialysis, MACE major adverse cardiovascular events, PTX parathyroidectomy

**Fig. 3:**
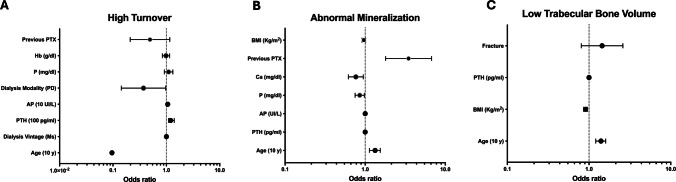
Multivariable analyses according to TMV status. Odds ratio for high bone turnover (**A**), adjusted for dialysis vintage, prior PTX, phosphate, and hemoglobin; abnormal mineralization (**B**), adjusted for BMI, PTH, and ALP; and low trabecular bone volume (**C**), adjusted for PTH and history of fractures

No biochemical parameter could be used as an index for detecting low trabecular volume, which was more likely to be present in older people with lower BMI and documented prior fractures. As shown in Fig. 3, multiple regression analysis revealed that low trabecular volume was independently associated with age and BMI in a model adjusted for PTH and history of fractures. High turnover was inversely associated with age and positively associated with PTH and ALP levels after adjustment for dialysis vintage, prior parathyroidectomy, serum phosphate, and hemoglobin. Abnormal mineralization was independently associated with age, phosphate, and calcium levels after adjustment for BMI, PTH, and ALP.

## Discussion

In this bone biopsy–centered analysis of patients with advanced CKD on dialysis, we demonstrate that low trabecular bone volume was commonly observed in our cohort and occurred across different patterns of ROD, including both high and low turnover states. Clinical and laboratorial results are able to assist with diagnosis of disturbed bone turnover or altered mineralization [4], but cannot anticipate reduced trabecular bone volume in CKD patients, which depends on imaging and/or histological assessment.

Bone turnover comprises resorption and formation occurring in bone remodeling units in cancellous and cortical bone, necessary to keep mineral balance and maintain bone strength. In this study, OF was the most prevalent ROD subtype, followed by AB and MBD. Regardless of ethnicity, higher bone turnover has been reported mainly in younger CKD populations [10]. We show that high turnover negatively correlates with age and is associated with higher levels of PTH and ALP. In fact, high-turnover disease is essentially the bone histological feature of CKD-related hyperparathyroidism, commonly detected among patients in this cohort, since 30% had PTH levels 9 times above the reference range [11].

Mineralization defects were identified in patients with OM, as a primary abnormality, or associated with high bone turnover, as mixed bone disease. In the present analysis, mineralization defects were more commonly observed in older patients, in those with lower levels of calcium and phosphate, as well as in those previously submitted to PTX. Prior studies have reported an association between PTX and subsequent worsening of bone mineralization in patients on dialysis, with evidence of deterioration up to one year after PTX [12]. In addition, hypocalcemia and hypophosphatemia are well-established contributors to vitamin D deficiency–related osteomalacia [13], and low phosphate levels have also been associated with impaired mineralization in the post-transplant scenario [14]. More studies focusing on the skeletal effects of different levels of calcium or phosphate are still needed.

Adynamic bone refers to absent or reduced bone turnover in the presence of normal mineralization, condition that has been associated with poor outcomes, such as mortality and progression of vascular calcification [15, 16]. However, it must be denoted that low turnover is associated with other frequent conditions that can increase mortality in CKD context, such as chronic inflammation, malnutrition, and diabetes. Further, low bone turnover state has become increasingly prominent not only in early CKD stages 2 to 3 [17], but also in patients on dialysis [6, 10], with impact on altered calcium homeostasis [18] and increased risk of vascular calcification [16]. Although it has evolved as the most abundant form of ROD, according to large bone biopsy registries [6, 10], in this analysis, adynamic bone was the second most common ROD, following OF, underscoring the high amount of patients with secondary hyperparathyroidism in this cohort. Indeed, different perceptions about the ROD frequency might be confounded by biopsy indication in populations with different backgrounds.

According to the World Health Organization and the National Institute of Health (NIH), osteoporosis is characterized by low bone mass and microarchitectural bone deterioration that results in bone fragility and increased risk of fracture. Notably, we demonstrated that the distribution of low trabecular bone volume varies across different forms of ROD. In contrast to a previous study [19], which reported no association between trabecular bone volume and ROD type, our analysis showed that most patients with AB also exhibited low trabecular volume. This finding highlights the coexistence of low bone turnover and reduced trabecular volume—both central components of the CKD-OP phenotype. Distinct findings might be explained by different baseline characteristics between both study populations. In addition to a smaller number of participants, the former analysis used PTH level > 1000 pg/ml as an exclusion criteria, withdrawing cases with severe hyperparathyroidism, which corresponds to 18% of the current study population.

Low BMI and advanced age are well-known risk factors for osteoporosis. Both CKD and aging negatively impact cortical and trabecular microarchitecture, affecting bone quality in different manners [20]. In line with this finding, low bone trabecular volume correlated with age and BMI as expected, but not with fractures, which may be also related to the high prevalence of patients with severe hyperparathyroidism in this cohort, not reflected by the trabecular compartment.

The prognostic assessment of bone status is influenced by which bone sites are under investigation. In addition to the retrospective design, a potential limitation of the study is the lack of data on cortical bone compartment. Indeed, cortical-rich sites have been pointed out as more relevant than trabecular sites in predicting survival and worse outcomes in CKD patients assessed by DXA [21]. Also, the study does not include information on levels of FGF-23 and calcitriol, and for most of the biopsies, only qualitative assessments have been performed, since quantitative analyses have been reserved for a subset of biopsies included in research protocols. An additional limitation is the fact that the indications for bone biopsies were not uniform, being justified for several reasons, either to assist nephrologists with diagnosis and CKD-MBD management, or as a step to enroll patients in research protocols, which limits the generalizability of our findings. However, the key advantage of this analysis was to combine clinical and laboratorial data to a large number of bone biopsies from a contemporary cohort of patients with CKD.

Metabolic bone disease diagnosis in CKD is complex. In contrast to histomorphometry, imaging techniques are unable to capture bone turnover, which, in turn, can be predicted by circulating biomarkers. However, we are aware that available biochemical parameters are limited to being used as surrogate biomarkers for bone histology in CKD. PTH recommended target by the K/DOQI guidelines, for instance, has been associated with a high incidence of low-turnover bone disease [22], and previous studies have demonstrated a U-shaped relationship between PTH and fracture risk [23, 24], highlighting that both very high and very low PTH levels may impair bone quality in CKD patients and increase fracture risk. Therefore, a combined diagnostic arsenal is the ideal approach for fracture risk prediction and therapeutic decision-making, and more studies using bone biopsy are needed to investigate the relationship between bone histomorphometry, mineral density and clinical outcomes, such as fractures and vascular calcification.

In conclusion, clinical characteristics were associated with distinct bone histomorphometric patterns in patients with CKD-associated osteoporosis. Younger patients undergoing hemodialysis with elevated PTH and ALP levels were more likely to exhibit high-turnover bone disease, whereas older and more frail patients with relatively low PTH levels were more likely to present with low bone turnover and trabecular volume. These findings may help identify patients with different underlying bone phenotypes and support a more personalized approach to the management of CKD-associated osteoporosis.

## Data Availability

The datasets utilized and/or examined in this study can be obtained from the corresponding author upon reasonable request.
